# Pharmacokinetics of neutron-irradiated meglumine antimoniate in *Leishmania amazonensis*-infected BALB/c mice

**DOI:** 10.1590/1678-9199-JVATITD-1446-18

**Published:** 2019-03-11

**Authors:** Samanta Etel Treiger Borborema, João Alberto Osso, Heitor Franco de Andrade, Nanci do Nascimento

**Affiliations:** 1 Center for Biotechnology, Nuclear and Energy Research Institute, São Paulo, SP, Brazil.; 2 Center for Parasitology and Mycology, Adolfo Lutz Institute, São Paulo, SP, Brazil.; 3 Center for Radiopharmacy, Nuclear and Energy Research Institute, São Paulo, SP, Brazil.; 4 Laboratory of Protozoology, São Paulo Tropical Medicine Institute, University of São Paulo (IMTSP/USP) São Paulo, SP, Brazil.

**Keywords:** cutaneous leishmaniasis, meglumine antimoniate, pharmacokinetics, biodistribution, antimony, radioisotope

## Abstract

**Background:**

Cutaneous leishmaniasis (CL) is a parasitic disease caused by the protozoan *Leishmania* spp. Pentavalent antimonial agents have been used as an effective therapy, despite their side effects and resistant cases. Their pharmacokinetics remain largely unexplored. This study aimed to investigate the pharmacokinetic profile of meglumine antimoniate in a murine model of cutaneous leishmaniasis using a radiotracer approach.

**Methods:**

Meglumine antimoniate was neutron-irradiated inside a nuclear reactor and was administered once intraperitoneally to uninfected and *L*. *amazonensis*-infected BALB/c mice. Different organs and tissues were collected and the total antimony was measured.

**Results:**

Higher antimony levels were found in infected than uninfected footpad (0.29% IA vs. 0.14% IA, p = 0.0057) and maintained the concentration. The animals accumulated and retained antimony in the liver, which cleared slowly. The kidney and intestinal uptake data support the hypothesis that antimony has two elimination pathways, first through renal excretion, followed by biliary excretion. Both processes demonstrated a biphasic elimination profile classified as fast and slow. In the blood, antimony followed a biexponential open model. Infected mice showed a lower maximum concentration (6.2% IA/mL vs. 11.8% IA/mL, p = 0.0001), a 2.5-fold smaller area under the curve, a 2.7-fold reduction in the mean residence time, and a 2.5-fold higher clearance rate when compared to the uninfected mice.

**Conclusions:**

neutron-irradiated meglumine antimoniate concentrates in infected footpad, while the infection affects antimony pharmacokinetics.

## Background

Cutaneous leishmaniasis (CL) is a group of diseases with different clinical manifestations ranging from small cutaneous nodules to gross mucosal tissue dissemination [1]. It is caused by the intracellular protozoan parasites of the genus *Leishmania* and is transmitted to humans via the bite of sandflies [2,3]. It is endemic in more than 70 countries and is widely distributed in the Americas, the Mediterranean basin, Asia, and Africa. About 75% of the global estimated CL cases are concentrated in ten countries: Afghanistan, Algeria, Brazil, Colombia, Costa Rica, Ethiopia, Iran, North Sudan, Peru and Syria [4].

Cutaneous leishmaniasis is not fatal, and ulcers can heal spontaneously, so treatment is utilized to accelerate a cure, reduce scarring, and prevent parasite dissemination (mucosal leishmaniasis) and relapse. Although progress has been made in assessing new therapeutic alternatives for leishmaniasis [5], the mainstay of treatment remains pentavalent antimonial agents in the form of sodium stibogluconate (Pentostam®) or meglumine antimoniate (MA, Glucantime®) administered parenterally or intralesionally [6]. However, these agents cause side effects; furthermore, some parasite strains are drug resistant, and their pharmacokinetic profiles remain poorly explored. Treatment failures could be due to inherent resistance of the parasite, an immunological defect in the host, or sub-therapeutic levels of antimony due to a lack of information on its pharmacokinetic properties [7-9].

The disease has enormous health, social, and economic impacts with significant morbidity, particularly in the tropical regions of the world [10]. The global burden of CL is exacerbated by the lack of vaccines, making safe and effective drugs imperative to its prevention and treatment [11]. The need for new drugs drives drug discovery and development research globally; however, these processes are very expensive and slow. It is very difficult to recover such investments from the sale of antiparasitic agents for neglected diseases such as leishmaniasis, resulting in insufficient funding and commitment from both public-sector agencies and the pharmaceutical industry [12].

Given these circumstances, a good understanding of the pharmacokinetics of the existing drugs could lead to a more successful strategy. The application of pharmacokinetic principles is one of the tools available for optimizing drug therapy, including drugs whose concentration-pharmacological response relationship is well established [13,14]. The pharmacokinetics of pentavalent antimonials have been reported in human patients [15,16], monkeys [17], and hamsters [18,19]. However, the available data are conflicting due to the different methodologies employed to measure antimony, the sample numbers, and the treatment schedules.

Few studies have reported the biodistribution and pharmacokinetic parameters of pentavalent antimonial drugs in tissues other than blood, and little is known about the profile between healthy individuals and those with CL [20]. It is important to know whether the infection can change the biodistribution pattern of a drug. Therefore, in continuation of the investigation of MA pharmacokinetic properties [21,22], the current study was undertaken to investigate its pharmacokinetic profile in *Leishmania* (*Leishmania*) *amazonensis*-infected mouse model using a radiotracer approach.

## Methods

### Animals

Female BALB/c mice (3 to 5-weeks old) were supplied by the animal breeding facility at the Faculty of Medicine of the University of São Paulo. Animals were maintained in sterilized cages in a controlled environment with free access to water and food. All of the animal procedures were performed with the approval of the Research Ethics Committee of the Tropical Medicine Institute of São Paulo, SP, Brazil (CEP-IMTSP 012/29/042008).

### Parasites

The *L.* (*L.*) *amazonensis* LV79 strain (MPRO/BR/72/M1841) promastigotes were cultivated in RPMI 1640 medium (Sigma-Aldrich Co, SP, Brazil) supplemented with 20% heat-inactivated fetal calf serum (Thermo Fisher Scientific, Inc, SP, Brazil) and 0.25% hemin (Sigma-Aldrich) in a BOD (biological oxygen demand) incubator at 24 °C.

### Production and analysis of irradiated meglumine antimoniate

Meglumine antimoniate (Glucantime®) was obtained from Sanofi-Aventis (SP, Brazil). Each 5 mL ampoule contained 1.5 g of MA, equivalent to 405 mg of pentavalent antimony (Sb^+5^), which represented approximately 27% of the total salt. Aliquots of MA (0.5-0.8 mL) were sealed in quartz ampoules and irradiated at the IEA-R1 nuclear reactor facility of the Nuclear andEnergy Research Institute - National Nuclear Energy Commission (IPEN - CNEN - SP/Brazil) using a thermal neutron flux of 0.8-1.0 × 10^12^ n/cm^2^/s for 10 min as previously described [21]. Antimony radioisotopes were produced by the nuclear reactions ^121^Sb(n,γ)^122^Sb and ^123^Sb(n,γ)^124^Sb. Radionuclidic purity was determined by γ-spectrometry using an HPGe detector coupled to the software Genie-PC (Canberra Inc., CT, USA). The concentrations of the radioactive compounds were measured via the same system after an efficiency calibration with standard ^60^Co, ^137^Cs, and ^152^Eu sources. The maintenance of MA’s biological properties was previously confirmed [21].

### Infection of BALB/c mice with *L*. (*L*.) *amazonensis*

To infect the animals, female BALB/c mice (n=40) were anesthetized by an association of ketamine (100 mg/kg) and xylazine (10 mg/kg) administered intramuscularly. The animals were subcutaneously infected in the footpad with 2 × 10^7^ stationary-phase promastigotes (6^th^ day of culture) of *L.* (*L.*) *amazonensis* in a final volume of 100 μL. To evaluate the disease progression, the mice were monitored weekly by observing the difference in thickness between the infected and contralateral uninfected footpads. The drug administration was initiated 50 days after infection, a time interval that allowed for the establishment of the disease, with the infection sites already swollen, and in some animals, ulcerating [23].

### Biodistribution of MA in uninfected and *L*. (*L*.) *amazonensis*-infected BALB/c mice

Two experimental groups of mice (22 ± 1 g) were used: (i) *L.* (*L.*) *amazonensis*-infected mice (n=40) and (ii) uninfected mice (n=40). The biodistribution of MA in uninfected animals previously reported by our research group [21,22] was utilized in the present study for comparison with that of infected animals. In each group, eight subgroups of five animals were randomly distributed. All animals received a single intraperitoneal injection of irradiated MA containing 0.081 mg of Sb^+5^/100 μL (administered dose was equivalent to 3.7 mg of Sb/kg) with the activity of 2.2 × 10^4^ Bq of ^122^Sb and 518 Bq μL of ^124^Sb [21]. The animals were euthanized 0.08, 0.25, 0.5, 1, 2, 5, 24 and 48 h after administration by cervical dislocation. Blood samples were collected from the retro-orbital plexus. The organs were collected, washed in distilled water to remove the blood, dried on filter paper and weighed. The whole infected and contralateral uninfected footpads were also removed and analyzed. The injected activity (IA) was measured in a NaI(Tl) scintillation counter (Cobra Auto-Gamma; Canberra Inc., CT, USA); the gamma energy range was established from 500-700 keV and counted for 1 min or until 1,000,000 counts per minute (cpm) was achieved. Skeletal muscle and blood volume were calculated as 40% and 7% of the body mass, respectively. The IA was established as 100% of the dose. The data were expressed as the percentage of IA per total organ or tissue (%IA), IA per gram of organ or tissue (%IA/g) or IA per milliliter of blood (%IA/mL).

### Pharmacokinetic analysis

A non-compartmental analysis of the blood concentration was performed using the software PK Solutions 2.0 (Summit Research Services, CO, USA). Peak concentrations in the blood (Cmax) and the time at which these concentrations were observed (Tmax) were determined from the concentration-time data. The classical trapezoidal rule was employed to compute the area under the drug concentration vs. time curve (AUC). The AUC was extrapolated to infinity by the addition of Clast/Kel, where Clast was the drug concentration in the last blood sample investigated and Kel was the terminal elimination rate constant. Kel was determined from the linear regression of the last three data points on each of the plots, and the blood half-lives (t_1/2_) were calculated as 0.693/Kel. The area under the first moment (AUMC) was determined using the same rules as for the AUC calculation. The mean residence time (MRT) was estimated as AUMC/AUC. The method of residuals (or curve-stripping) was applied to define the underlying exponential terms that best describe the current concentration-time data set. This procedure determines the pharmacokinetic parameters of half-life, rate and concentration intercept for each phase of the blood level curve [22].

### Statistical analysis

Pharmacokinetic parameters were derived from the mean concentrations of 5 animals at each time point. For the biodistribution data, the mean ± standard deviation (SD) of the measurements from 5 animals at each time point are shown. Data were analyzed using the software GraphPad Prism 5.0 (Prism Software, Irvine, CA). Each set of results was first checked for normal distribution using Kolmogorov-Smirnov, D’Agostinho and Pearson, and Shapiro-Wilk tests. Normally distributed data were analyzed through the unpaired two-tailed Student’s *t* test to compare the significance between uninfected and infected mice. Differences with p values of < 0.05 and < 0.0001 were considered statistically significant.

## Results

Meglumine antimoniate was administered once intraperitoneally to both uninfected and *L.* (*L.*) *amazonensis*-infected BALB/c mice to compare its biodistribution and pharmacokinetics in different organs. The total antimony was measured by counting the gamma emission of the irradiated MA.

**Figure 1:**
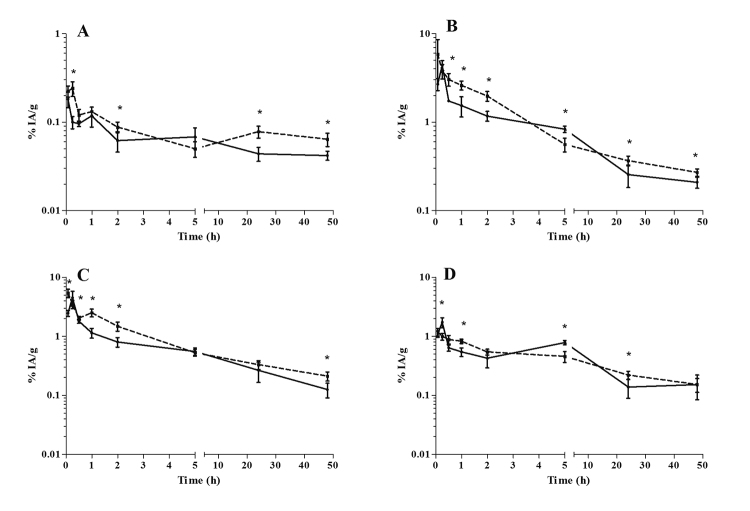
Antimony biodistribution in uninfected [22] and *L.* (*L.*) *amazonensis*-infected BALB/c mice after intraperitoneal administration of meglumine antimoniate. **A**: brain; **B**: heart; **C**: lung; **D**: muscle. Continuous line: infected mice; dotted line: uninfected mice. Data are expressed as the mean ± standard deviation (n= 5/time) of the percentage of injected activity (IA) per gram. The significance of differences between uninfected and infected mice was calculated by Student’s *t* test. * p < 0.05.

The antimony concentrations in the brain, heart, lungs, and muscle are displayed in Fig. 1. The infected and uninfected animals showed a low uptake of antimony in the brain, with approximately 0.18 % IA/g and 0.22% IA/g (p = 0.0899) at 0.08 h, and 0.04 % IA/g and 0.06% IA/g (p= 0.0070) at 48 h, respectively (Fig. 1A). In the heart, the antimony activity gradually decreased from approximately 4.4 % IA/g and 3.78% IA/g (p = 0.1936) at 0.25 h, and 0.21 % IA/g and 0.27% IA/g (p = 0.0160) at 48 h in infected and uninfected mice, respectively (Fig. 1B). In the lungs, the respective antimony activities in infected and uninfected mice were 4.51% IA/g and 3.5% IA/g (p = 0.1575) at 0.25 h, and 0.12% IA/g and 0.21% IA/g (p = 0.0065) at 48 h (Fig. 1C). In the skeletal muscle, the antimony activity gradually decreased in both animal groups from 1.1% IA/g at 0.08 h to 0.15% IA/g at 48 h (Fig. 1D).

**Figure 2:**
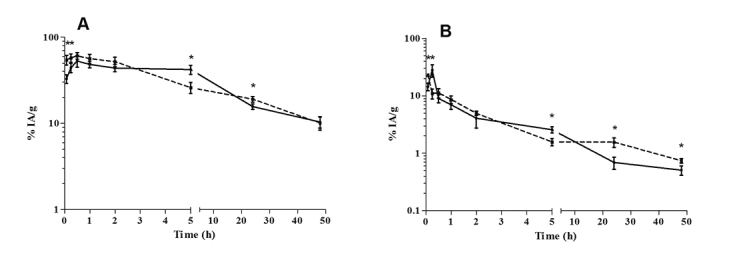
Antimony biodistribution in uninfected [22] and *L.* (*L.*) *amazonensis*-infected BALB/c mice after intraperitoneal administration of meglumine antimoniate. **A**: liver; **B**: spleen. Continuous line: infected mice; dotted line: uninfected mice. Data are expressed as the mean ± standard deviation (n= 5/time) of the percentage of injected activity (IA) per gram. The significance of differences between uninfected and infected mice was calculated by Student’s *t* test. * p < 0.05.

The antimony concentrations in the liver and spleen are shown in Fig. 2. The pharmacokinetic parameters indicated that the liver had rapidly taken up the antimony by 0.5 h, with infected mice having a lower Cmax (53% IA/g) than the uninfected mice (61% IA/g) (p = 0.0897). The analysis demonstrated a prolongation of the antimony MRT to 41 h in the uninfected mice compared to just 30 h in the infected mice. The AUC did not differ statistically between the infected and uninfected animals (1409% IA.h/g vs. 1463% IA.h/g). The higher AUC value is explained by the accumulation and retention of antimony in the liver combined with slow clearance rate, reaching a level of 10% IA/g at 48 h, in both groups (Fig. 2A).

The antimony uptake into the spleen was faster in the uninfected than in the infected mice, with Cmax values of 23% IA/g at 0.08 h and 28% IA/g at 0.25 h, respectively. The corresponding AUCs were 87% IA.h/g (infected) and 126% IA.h/g (uninfected). The analysis demonstrated a prolongation of the antimony MRT to 44 h in the uninfected mice and 21 h in the infected mice, with no statistically significant differences. The antimony was gradually eliminated from the spleen, reaching respective levels at 48 h of 0.51% IA/g and 0.74% IA/g (p = 0.0073) in infected and uninfected mice (Fig. 2B).

**Figure 3:**
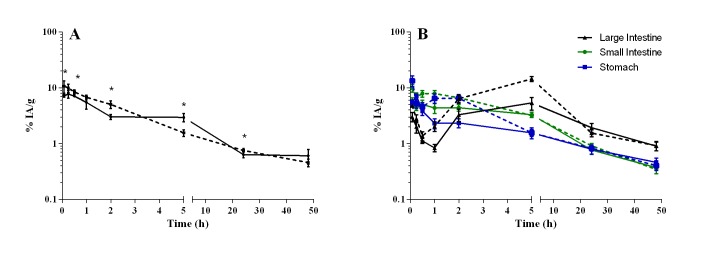
Antimony elimination pathways in uninfected [22] and *L.* (*L.*) *amazonensis*-infected BALB/c mice after intraperitoneal administration of meglumine antimoniate. **A**: kidney; **B**: gastrointestinal tract. Continuous line: infected mice; dotted line: uninfected mice. Data are shown as the mean ± standard deviation (n= 5/time) of the percentage of injected activity (IA) per gram. The significance of differences between uninfected and infected mice was calculated by Student’s *t* test. * p < 0.05.

The kidneys (Fig. 3A) rapidly eliminated antimony. The elimination occurred faster in the uninfected mice (Cmax of 11% IA/g at 0.08 h) than in those infected (Cmax of 8% IA/g at 0.25). A biphasic elimination profile was observed, which corresponded to a fast antimony elimination phase (E phase) for up 2 h after administering the drug, followed by a slow E phase that lasted at least until 48 h, with levels dropping to as low as 0.5% IA/g, in both animal groups. A fraction of antimony was also absorbed and distributed by the gastrointestinal tract (Fig. 3B), with elimination occurring through hepatobiliary excretion after processing in the liver, eventually reaching the intestinal lumen. High absorption levels occurred in the stomach of the uninfected mice by 0.08 h (13% IA/g) and by 0.25 h in the infected mice (7% IA/g). The antimony concentration decreased from 1.6% IA/g at 5 h post-injection to 0.4% IA/g at 48 h, in both groups. Similarly, in the small intestine, the antimony concentration decreased from 3% IA/g at 5 h to 0.3% IA/g at 48 h. The moment the small intestine showed a significant elimination of antimony, the large intestine manifested an increased concentration, representing the passage of the drug metabolized by the liver to the small intestine and then to the large intestine. The peak of the fast E phase in the large intestine occurred at 5 h, with 14.4% IA/g and 5.3% IA/g (p = 0.0004) for the uninfected and infected mice, respectively. This was followed by a slow E phase until at least 48 h, with levels dropping to 0.9% IA/g, in both groups.

**Figure 4:**
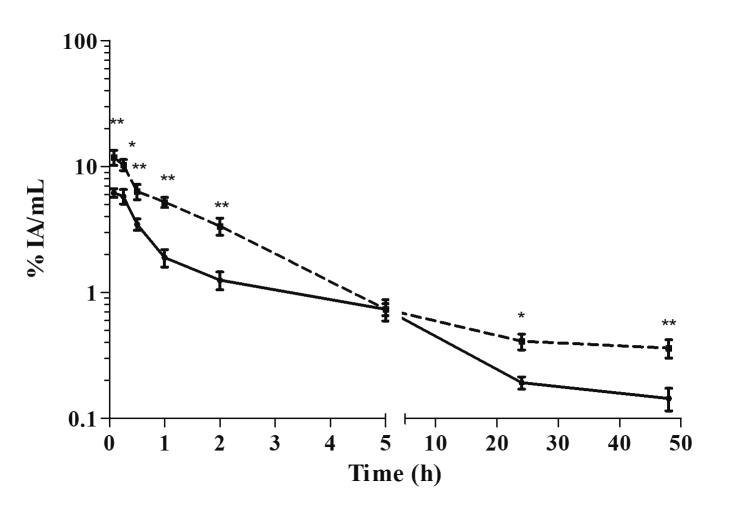
Blood pharmacokinetics of antimony in uninfected [22] and *L.* (*L.*) *amazonensis*-infected BALB/c mice after intraperitoneal administration of meglumine antimoniate. Continuous line: infected mice; dotted line: uninfected mice. Data are expressed as the mean ± standard deviation (n= 5/time) of the percentage of injected activity (IA) per milliliter of blood. The significance of differences between uninfected and infected mice was calculated by Student’s *t* test. * p < 0.05 and ** p < 0.0001.

The blood antimony concentration vs. time data collected after the intraperitoneal injection are displayed in Fig. 4. The means of the pharmacokinetic parameters corresponding to the analysis of the antimony blood concentration are summarized in Table 1. Antimony was rapidly absorbed by 0.08 h, with mean Cmax values of 11.8% IA/mL and 6.2% IA/mL (p = 0.0001) for the uninfected and infected mice, respectively. The distribution phases occurred simultaneously, with a faster E phase until 5 h after drug administration. Thereafter, the slower E phase occurred until at least 48 h, when it had reached 0.36% IA/mL and 0.14% IA/mL (p = 0.00001) for the uninfected and infected mice, respectively. The infected mice had a 2.5-fold lower AUC, a 2.7-fold reduction in the MRT, and a 2.5-fold higher CL compared to the uninfected mice.

**Table 1:** Mean pharmacokinetic parameters in the blood of uninfected (n= 5/time) and *L.* (*L.*) *amazonensis*-infected BALB/c mice (n= 5/time) following intraperitoneal administration of meglumine antimoniate.

Parameter	*L.* (*L.*) *amazonensis*-infected mice	Uninfected mice^a^
C_max_ (%IA/mL)	6.2*	12.7
T_max_ (h)	0.08	0.08
t_1/2_ E phase (h)	18.85*	48,91
t_1/2_ D/A phase (h)	0.92*	5.85
AUC_0-∞_ (%IA.h/mL)	25.1*	62.8
AUMC (%IA.h^2^/mL)	524.1*	3493.9
MRT (h)	20.8*	55.7
CL (mL/h)	3.97*	1.59

C_max_, peak plasma concentration; T_max_, time to C_max_; t_1/2_, plasma half-life; E phase, elimination phase; D/A phase, distribution or absorption phase; AUC, area under the concentration-time curve; AUMC, area under the first moment curve; MRT, mean residence time; CL, total clearance. The significance of differences between uninfected and infected mice was calculated by Student’s *t* test. * p<0.05. ^a^[22]

**Figure 5:**
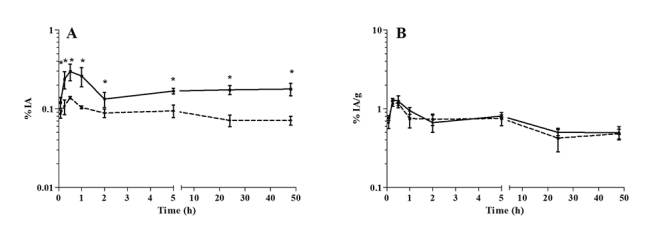
Biodistribution of antimony in uninfected [22] and contralateral *L.* (*L.*) *amazonensis*-infected footpads of BALB/c mice after intraperitoneal administration of meglumine antimoniate. **A**: percentage of injected activity (IA) in total; **B**: percentage of injected activity (IA) per gram. Continuous line: infected; dotted line: uninfected. Data are shown as the mean ± standard deviation (n= 5/time). The significance of differences between uninfected and infected mice was calculated by Student’s *t*-test. * p < 0.05.

Considering the whole footpad, the mean Cmax for antimony in the infected footpad (0.29% IA) was 2.2-fold higher than that in the contralateral uninfected footpad (0.14% IA) and was achieved at 0.5 h (p = 0.0057; Fig. 5A). At 2 h post-injection, the antimony concentration had decreased to 0.13% IA and 0.09% IA (p = 0.0273) in the infected and uninfected footpads, respectively. Moreover, the drug was retained at a higher level in the infected than in the uninfected footpad, and the levels were sustained through the latest period, reaching 0.18% IA and 0.07% IA (p = 0.0013) at 48 h, respectively. No statistically significant differences were found in the footpads’ antimony absorption as observed relative to the % IA/g in the tissue (Fig. 5B).

## Discussion

This work provides extensive data on the pharmacokinetics of meglumine antimoniate in *L. amazonensis*-infected BALB/c mice. Studying the biodistribution of MA is easier when the drug is radioactive, but there are no antimony radioisotopes commercially available. As we previously described, irradiating the MA was the best choice by which to support this goal in *in vivo* studies [21,22,24,25]. The advantage of this procedure is that neutron irradiation of the stable antimony isotopes present in the MA formulation enables the detection and quantification of total antimony, but without distinguishing between the pentavalent and trivalent antimony species. We also found that the irradiated MA provided antileishmanial activity similar to that of the non-irradiated MA in both *in vitro* and *in vivo* evaluations. These findings indicate that the biological activity of MA was preserved after the irradiation procedure and excluded the hypothesis that irradiated MA was converted into trivalent antimony [21]. The possible change in the polymerization state of meglumine antimoniate upon neutron irradiation may affect the pharmacokinetics of antimony [26]; further studies should be performed to evaluate this issue.

In our study, based on the antimony uptake, the levels in the organs can be classified as low (<1%) in the brain; intermediate (>1-10%) in the heart, stomach, small intestine, muscle, infected and uninfected footpads, lungs, kidneys, and blood; and high (>10%) in the spleen, liver and large intestine. Compared to uninfected mice, the *L. amazonensis*-infected mice showed lower concentrations of antimony with smaller tissue persistence in the liver and spleen. These alterations may be associated with the tissue damage caused by the infection. *L. amazonensis*-infected mice have shown an induction of a strong inflammatory response in the skin, with possible occurrence of parasitic migration to secondary organs and consequent tissue injury [27,28]. Previous works have demonstrated that *L. amazonensis* may cause the localized, diffuse or mucosal clinical forms of leishmaniasis and is capable of dissemination to internal organs, causing the visceral form [29].

The infiltration into the skin by the *Leishmania* parasites and the resulting damage to both tissue and blood vessels highlight the importance of examining the impact of this infection on the pharmacokinetics of the antimony in the lesion/skin. Understanding the uptake and disposition of antimony in the affected skin is vital for optimizing the dosage regimens when treating CL [30]. In the current study, we found that the infected footpad concentrated more antimony compared to the uninfected footpad in the same animal. The antimony was also retained longer in the footpad than in the blood. A similarly long retention of antimony by the skin has also been reported in patients treated for CL with meglumine antimoniate [31,32]. However, Al Jaser and coworkers [30] observed no significant differences in any of the pharmacokinetic parameters, including the AUC_skin_/AUC_blood_ ratio, between the affected and normal skin, suggesting that the infection had no impact on the antimony pharmacokinetics in the skin.

Comparing the results for the footpad with that for the blood in infected animals, the Cmax in the blood was approximately 5-fold higher, while the Tmax was achieved 3 times faster. However, the drug was retained in the footpad longer and its elimination was slower. The mean concentration of antimony in the footpad was lower than that in blood for the first 5 h, but increased thereafter. The same profile has been observed in *L. donovani*-infected hamsters following the administration of sodium stibogluconate [18] and in patients with CL [30]. Elevated tissue drug levels may be associated with increased vascular permeability and macrophage infiltration in the infected skin, as reported after treatment with liposomal amphotericin B [33,34]. However, one limitation of our present study is its inability to distinguish antimony accumulation between the skin/lesion and the whole footpad due to, for example, swelling and accumulation of blood in the footpad. In order to overcome this limitation, further studies should be conducted using a mouse model with infection at the tail base [35].

It is currently unknown to what degree our observations of skin/lesion accumulation of antimony in the *L. amazonensis*-BALB/c model are translatable to human CL, but elucidation of preclinical pharmacokinetics should improve the use and development of antileishmanial drugs. The increase of antimony uptake by infected footpad after systemic administration may provide an understanding of its pharmacokinetics, which could assist in rationalizing and optimizing treatment regimens, especially in combining multiple antileishmanial drugs in an attempt to increase efficacy and shorten treatment duration [14]. Furthermore, these findings may support further studies by enabling comparison with the pharmacokinetics of the antimony injected intralesionally, an alternative form of treatment [36,37].

The kidney and intestine uptake data support the hypothesis of two elimination pathways for antimony, initial elimination through renal excretion followed by biliary excretion. We previously demonstrated in *L. infantum*-infected mice that antimony was absorbed from the gastrointestinal tract and entered the liver, where it was metabolized before reaching the rest of the body [21,22]. The compound permeates from the blood into the hepatocytes, where it is metabolized by a variety of enzymatic reactions. The metabolites and a portion of the unchanged compound can be extracted into the bile (“biliary extraction”), which is stored in the gallbladder and excreted into the intestine, where they are eliminated in the feces. The metabolites and the unchanged compound can also exit from the hepatocytes into the blood and be extracted by the kidney into the urine [38].

During biotransformation reactions, original drugs are converted into more polar metabolites by oxidation, reduction or hydrolysis, and the resulting metabolites can be more active than the original molecule (prodrug) [39]. Thus, pentavalent antimonials would behave as a prodrug that is reduced within the organism into the more toxic and active trivalent antimonial [40]. Thiols can act as reducing agents in this conversion. Pentavalent antimony is reduced *in vivo* within *Leishmania* parasites by trypanothione and by cysteine and cysteinyl-glycine within the acidic compartments of mammalian cells; other parasite-specific enzymes such as thiol-dependent reductase and/or antimoniate reductase may be involved [41].

The biliary tree is known to be a major route of excretion for trivalent antimonial drugs, but for the pentavalent drugs, the kidney is the major excretion route, with the biliary tree acting as only a minor route [42,43]. Gyurasics and coworkers [44] demonstrated that the hepatobiliary transport of trivalent antimony is dependent on glutathione (GSH), and hypothesized that antimony is transported from liver cells into the bile canaliculi as unstable GSH complexes from which the metal is released, reabsorbed into the hepatocytes, and re-excreted, generating additional transport of GSH into the bile.

Our findings support the hypothesis that the kidneys first eliminate pentavalent antimony, while the intestines also eliminate a fraction of the drug. The slow terminal elimination phase by both routes may be related to the conversion of pentavalent antimony into trivalent antimony. There is a lack of reports regarding the biodistribution of pentavalent antimonials in the organs involved in enterohepatic elimination, particularly the intestines and gallbladder, highlighting the need for further studies to clarify the pharmacokinetics.

The clearance of the antimony from circulation also had a biphasic character. The low antimony level after 5 h can be explained by both its rapid clearance by the liver and its rapid renal elimination. The small fraction of antimony that is more slowly eliminated may be related to its accumulation in the body during treatment.

A similar pharmacokinetic profile has been proposed in CL patients [16] and in visceral leishmaniasis patients treated with sodium stibogluconate [45]. The first kinetic compartment is a central one that includes the blood volume into which the drug is absorbed and from which the drug is excreted into the urine. The second compartment may be a peripheral one into which the drug is distributed, or it may be related to the *in vivo* conversion of pentavalent to trivalent antimony [45]. Trivalent antimony becomes a major antimony species in the plasma during the terminal slow elimination phase of pentavalent antimonial drugs, supporting the hypothesis that it is reduced to trivalent antimony within the cells, where it is further released at a slow rate [17].

Due to very limited treatment options for leishmaniasis patients, optimization of current drug dosages and drug combinations is of utmost importance. The current study provides the pharmacokinetic properties of antimony in a cutaneous leishmaniasis model that might be useful to improve clinical outcomes for choosing the appropriate drugs, or combination thereof, and their dosages. Therefore, several new drug combinations are currently being tested to improve the efficacy of antileishmanial therapies, and may shorten treatment duration [8,46].

A wide variation in observed antimony tissue concentrations among different reports may influence antimony efficacy in CL treatment. The knowledge of pharmacokinetic parameters could drive studies to relate skin exposure to treatment outcome. Our data may support exposure-response studies linking the pharmacokinetics of antileishmanial drugs to treatment outcome. Moreover, future studies should also investigate the pharmacokinetics of antileishmanial drugs especially in vulnerable patient populations such as pediatric leishmaniasis patients and with co-infections. Evaluation of pharmacokinetics in preclinical models is important for ascertaining optimal clinical use and providing lessons for drug development. Available pharmacokinetic data could allow for optimizing the chemotherapeutic regime to extend its use and reduce its failures [13].

## Conclusions

Neutron-irradiated meglumine antimoniate is more highly concentrated in infected footpad for a longer duration than in uninfected footpad. The *Leishmania* infection has an impact on the pharmacokinetics and penetration of antimony into the footpad. This work emphasizes the importance of antimony’s pharmacokinetic profile in finding better therapeutic protocols as to its dosage, administration interval, and the duration of therapy.

## Abbreviations

Not applicable.
